# Molecular mechanisms behind the accumulation of ATP and H_2_O_2_ in citrus plants in response to ‘*Candidatus* Liberibacter asiaticus’ infection

**DOI:** 10.1038/hortres.2017.40

**Published:** 2017-08-23

**Authors:** Marco Pitino, Cheryl M Armstrong, Yongping Duan

**Affiliations:** USDA-ARS, US Horticultural Research Laboratory, 2001 S. Rock Road, Fort Pierce, 34945 FL USA

**Keywords:** Biotic, Plant physiology

## Abstract

*Candidatus *Liberibacter asiaticus (Las) is a fastidious, phloem-restricted pathogen with a significantly reduced genome, and attacks all citrus species with no immune cultivars documented to date. Like other plant bacterial pathogens, Las deploys effector proteins into the organelles of plant cells, such as mitochondria and chloroplasts to manipulate host immunity and physiology. These organelles are responsible for the synthesis of adenosine triphosphate (ATP) and have a critical role in plant immune signaling during hydrogen peroxide (H_2_O_2_) production. In this study, we investigated H_2_O_2_ and ATP accumulation in relation to citrus huanglongbing (HLB) in addition to revealing the expression profiles of genes critical for the production and detoxification of H_2_O_2_ and ATP synthesis. We also found that as ATP and H_2_O_2_ concentrations increased in the leaf, so did the severity of the HLB symptoms, a trend that remained consistent among the four different citrus varieties tested. Furthermore, the upregulation of ATP synthase, a key enzyme for energy conversion, may contribute to the accumulation of ATP in infected tissues, whereas downregulation of the H_2_O_2_ detoxification system may cause oxidative damage to plant macromolecules and cell structures. This may explain the cause of some of the HLB symptoms such as chlorosis or leaf discoloration. The findings in this study highlight important molecular and physiological mechanisms involved in the host plants’ response to Las infection and provide new targets for interrupting the disease cycle.

## Plant pathology: The juice on citrus greening disease

The physiological processes occuring in citrus plants suffering from citrus greening disease are at last becoming clear. This devasting disease, also known as Huanglongbing, is caused by the enigmatic bacterium *Candidatus* Liberibacter asiaticus (‘Las’). Las infects all citrus species, and no resistant cultivars are known. It has so far evaded laboratory culture, but a team at USDA-ARS in Fort Pierce, Florida, led by Yongping Duan, have now characterized how it manipulates host cells. Las-infected plants accumulate hydrogen peroxide (H_2_O_2_), which destroys plant cells but which Las can detoxify. Las also causes its host to generate increased levels of adenosine triphosphate (ATP), the energy currency of cells, which is translocated to the bacterium, fueling the infection. ATP and H2O2 may provide useful markers for infection, as well as targets for potential disease control strategies.

## Introduction

*Candidatus* *Liberibacter asiaticus* (Las) is the predominant species of *Liberibacter* associated with huanglongbing (HLB), a disease that is currently considered the most devastating among citrus diseases worldwide.^1,2^ Las pathogen is fastidious, vector-borne,^3^ and restricted to the phloem.^1,4^ Despite its significantly reduced genome (1.26 Mb),^5,6^ Las has been shown to attack all citrus species and citrus hybrids in the *Citrus* genus,^7,8^ with no immune cultivars documented to date. The symptoms of HLB observed in Las-infected plants are thought to result from unique host–pathogen interactions. Typical HLB symptoms include vein yellowing, yellow shoots, leaf curl with vein corking, asymmetric blotchy mottle on leaves and dieback.^9^ HLB weakens the root system, increases early fruit abscission, and ultimately causes high tree mortality.^8^ Identification of the host responses to the pathogen is critical for understanding disease development overall and may be exploited in the formulation of efficient disease management practices.^10^

When a pathogen attacks, an array of defense mechanisms become activated within the host plant including both basal defense and gene-for-gene resistance. Jones and Dangl^11^ conceptualized the plant immune system using a zig-zag model with three phases. During the first phase of the infection process, microbe-associated molecular patterns (MAMPs) or pathogen-associated molecular patterns (PAMPs), and plant degradation products known as damage-associated molecular patterns (DAMPs), are generated.^12^ These products are recognized by the host’s pathogen recognition receptors (PRRs)^13^ resulting in PAMP-triggered immunity (PTI). Activation of the PTI response leads to an influx of extracellular Ca^2+^ in the cytosol (Ca^2+^ burst),^14,15^ which regulates respiratory burst oxidase homolog (Rboh).^16,17^ Rboh is primarily regulated through post-translational modifications induced by Ca^2+^,^18^ an intracellular messenger,^19^ and is involved in the production of reactive oxygen species (ROS) for physiological and developmental purposes.^20^ Subsequent to the production of membrane-impermeable superoxide O_2_^−^ in the apoplast, the O_2_^−^ is converted into hydrogen peroxide (H_2_O_2_) by superoxide dismutases (SOD).^21^ H_2_O_2_ production leads to a broad-spectrum resistance against microbes because of it is membrane permeability, which allows it to enter into the cytosol and migrate into different compartments thereby facilitating its signaling functions.^22^ In the second phase, successful pathogens deploy effectors, which contribute to pathogen’s virulence by interfering with the PTI response of the plant.^23^ In phase three, plants may recognize these effector molecules by intracellular receptors (R proteins), resulting in effector-triggered immunity (ETI). The ETI response is a more rapid and robust response than PTI and consists of higher levels of H_2_O_2_ production and increased callose deposition.^24^ Pathogens may escape ETI through loss or mutation of recognized effectors or by suppressing ETI using additional novel effectors.^25^

Virulent pathogens that avoid host recognition induce a low-amplitude first phase response in plants and in doing so produce ROS, which may result in a strengthening of host cell walls,^26^ lipid peroxidation and membrane damage.^27^ Active H_2_O_2_ production occurs primarily at the apoplastic space and is a prerequisite for both oxidative burst-mediated signaling related to the hypersensitive response in addition to being an integral part of plant development and cell death.^28^ The enzymatic antioxidants, including ascorbate peroxidases, glutathione, superoxide dismutase and catalases, maintain ROS homeostasis in different cellular compartments, but the presence of pathogens can alter this balance.^29^ Although ROS production is usually a method used to defend the plant against attack, for some pathogens ROS production can be beneficial.^30^ For example, interference with the chlorophyll degradation pathway results in an over-accumulation of ROS, which increases plant cell death and thereby benefits the pathogen by increasing the availability of nutrient.^31^

Even though a multitude of studies have been performed that investigate the plant–microbe interactions between citrus plants and Las, HLB is still not completely understood. Transcriptome analysis has been used to successfully identify how Las infection influences gene expression in citrus plants on a global scale.^9,32,33^ In particular, extensive changes in gene expression were identified for major biological processes such as stress responses, signal transduction, transport, cell organization and carbohydrate metabolism.^34^ From a bacterial prospective, several proteins have been identified as important for virulence and growth. Previous studies have indicated that the mitochondria^35^ and chloroplast^36^ are potential targets of Las protein effectors. Mitochondria and chloroplasts are responsible for the synthesis of adenosine triphosphate (ATP), the major energy currency molecule of the cell. They also have a critical role in plant immune signaling for both PTI and ETI, and in ROS production.^15^ Moreover, a functional ATP translocase, which allows for the import of ATP/ADP directly from its host cells, has been identified in Las.^37^ Bacteria that possess this transporter can act as ‘energy parasites’ and import ATP directly from their hosts. Recently, a peroxidase, an extracellular and functionally active H_2_O_2_ scavenging enzyme, was identified as having an important role in Las survival by providing an essential defense against ROS generated by the infected plant cell.^38^ Taken together, these data could indicate that Las effectors may either directly or indirectly manipulate the mitochondria and chloroplasts to modify ATP production by altering the redox homeostasis in an effort to promote its growth.

In this work, we studied host responses of citrus to Las infection and investigated the HLB symptoms in relation to H_2_O_2_ production and ATP accumulation. We analyzed four Las-infected and non-infected citrus varieties [grapefruit (*Citrus*×*paradisi* ‘Duncan’), sweet orange (*Citrus*×*sinensis* ‘Valencia’), sour orange (*Citrus*×*aurantium* ‘Karun Jamir’) and lemon (*Citrus*×*limon* ‘vulcan’)] for ATP production, and the expression of genes involved in the formation and detoxification of H_2_O_2_, in particular, we analyzed the gene expression of respiratory burst oxidase homologs *RBOH*,^20,39^ the enzymatic antioxidants ascorbate peroxidase *APX*, catalase *CAT*, superoxide dismutase *SOD* ^40–44^ for the H_2_O_2_ and ATP synthase beta subunit from chloroplast *CATPb* and mitochondria *MATPb* for ATP. Pathways involving H_2_O_2_ were chosen because H_2_O_2_ not only represents one of the major and most stable end products of ROS production but it has also been shown to regulate basic acclamatory response, defense and developmental processes in plants.^45,46^ Our results highlight the molecular and physiological processes associated with HLB disease progression.

## Materials and methods

### Citrus leaves and Las titer

The leaves used for the analysis were collected from four different citrus varieties, grapefruit (*Citrus×paradisi,* ‘Duncan’), sweet orange (*Citrus*×*sinensis,* ‘Valencia’), sour orange (*Citrus*×*aurantium,* ‘Karun Jamir’) and lemon (*Citrus*×*limon,* ‘Vulcan’). Plants were grown in the screened US Horticultural Research Laboratory greenhouse in Fort Pierce, FL, USA. For each variety tested, 20 individual 2-years-old healthy plants were graft-inoculated via side-grafting with 3–4 cm Las-positive lemon bud sticks. Sour orange and grapefruit, were grown from seed, whereas sweet orange and lemon were grown on sour orange rootstock. All plants were housed at the US Horticultural Research laboratory where they were irrigated and fertilized every 3 weeks. Real-time PCR values corresponding to Las titer were used in addition to the presence of disease symptoms to confirm that plants are either healthy or Las-infected.^47^ Total genomic DNA was extracted from Las-infected citrus leaves as described previously.^36,48^

**Figure 1:**
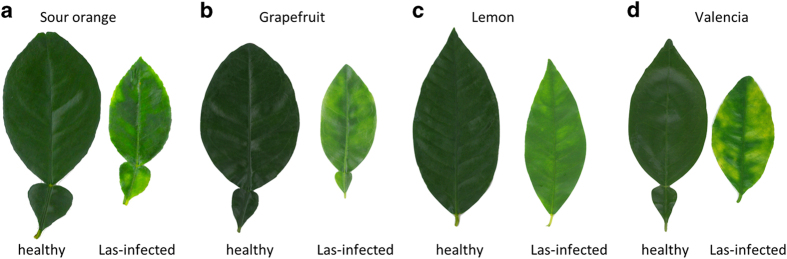
Las-infected and non-infected sample leaves used in the study. DNA was extracted from the midribs, whereas the left and right side of the leaf were used in the leaf discs assays for ATP and H_2_O_2_ production and for the transcriptional profiling performed via RT-qPCR analysis on the following citrus varieties: (**a**) sour orange, (**b**) grapefruit, (**c**) lemon and (**d**) sweet orange.

**Figure 2:**
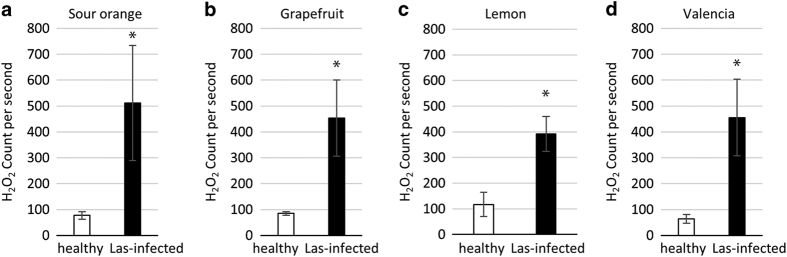
H_2_O_2_ levels increase in Las-infected leaf discs. H_2_O_2_ levels were higher in Las-infected tissue (black bars) compared with the non-infected samples (white bars) in four different citrus varieties. Nine leaf discs using three leaves per sample were used for the analysis. Each bar represents the mean of 10 replicates, with the error bars representing the standard deviation. Asterisk indicates significant differences in Las-infected leaves compared with the uninfected (Student’s *t*-test, *P*<0.05).

**Figure 3:**
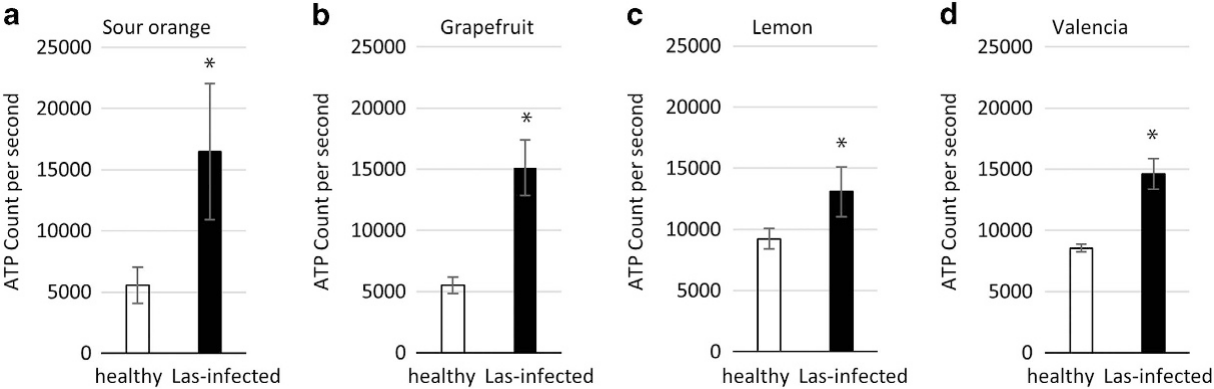
Increased ATP level in Las-infected leaf samples. ATP levels are higher in Las-infected tissue (black bars) compared with the non-infected tissues (white bars) in four different citrus varieties. Six leaf discs using three leaves per sample were used for this analysis. Each bar represents the mean of 10 replicates, with the error bars representing the standard deviation. Asterisk indicates significant differences in Las-infected leaves compared with the uninfected (Student’s *t*-test, *P*<0.05).

**Figure 4:**
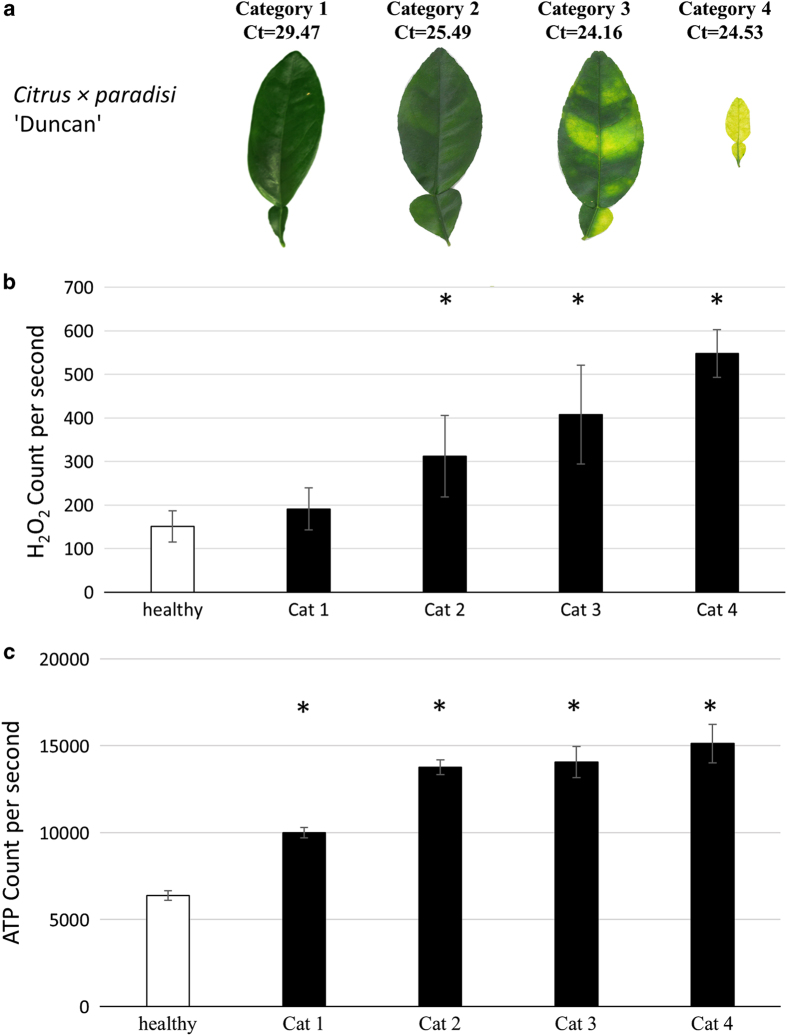
ATP and H_2_O_2_ levels directly correlate with disease severity in citrus leaves. (**a**) HLB symptoms on grapefruit leaves were classified into four different categories based on the severity of disease, ranging from mild to severe. Grapefruit leaves showed an increase in (**b**) basal H_2_O_2_ levels and (**c**) ATP levels as symptom severity increased. Each bar represents the mean of 10 replicates, with the error bars representing the standard deviation. Asterisk indicates significant differences in Las-infected leaves compared to uninfected (Student’s *t*-test, *P*<0.05).

**Figure 5:**
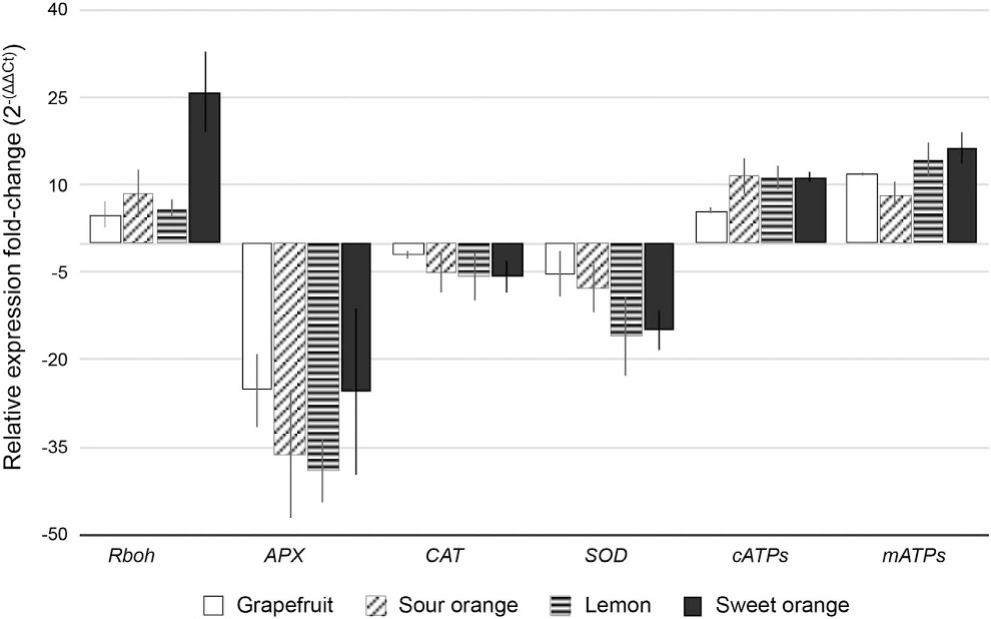
Differential regulation of genes in four Las-infected citrus varieties. The ROS detoxification genes *APX*, *CAT* and *SOD* were downregulated in Las-infected tissue from sour orange, grapefruit, lemon and sweet orange, whereas *RBOH* and the ATP synthase beta-subunits from chloroplast and mitochondria were upregulated in these same tissues.

To determine the Las bacterium titer, symptomatic and healthy leaves were collected from the four citrus species (Figure 1) and tested using TaqMan qPCR 16S rDNA-based TaqMan primer-probe^41^ on leaf midrib tissues. TaqMan real-time PCR amplifications, including the negative and positive controls, were performed in an Eppendorf Mastercycler realplex 4 PCR System (Eppendorf, Hamburg, Germany) using primers HLBasf, HLBr and probe HLBp targeting the 16S rDNA of Las.^49^ Overall, 15 μl qPCR reaction mixtures were used, which contained 7.5 μl TaqMan Fast Universal PCR Master Mix (Applied Biosystems, Carlsbad, CA, USA), 250 nm each primer, 150 nm probe and 100 ng template DNA. The PCR program started with a denaturation step of 95 °C for 30 s followed by 40 cycles of 95 °C for 5 s and 60 °C for 30 s). Only the symptomatic leaves which tested Las-positive with similar Ct values ~25 were then used for the measurement of the following: H_2_O_2_ (Figure 2), ATP (Figure 3) and RT-qPCR for gene transcript levels (Figure 5). We also identified and classified three different HLB symptom categories using the most characteristic symptoms including blotchy mottle and small yellow leaves (Figure 4). Ct values were used to analyze the different symptomatic leaves for Las bacterium titer, the nine leaf samples tested with an average of Ct value ~25 were used for further analyses.

### Measurement of H_2_O_2_ concentration via fluorescence

H_2_O_2_ detection was conducted as described in previous studies with the following minor modifications.^36,50–52^ Briefly, citrus leaf discs were collected from either the Las-infected citrus trees or non-infected trees using a circular 5 mm diameter cork borer. Ten different replicates were performed where each replicate, consisting of a total of nine leaf discs collected from three different symptomatic leaves of a single plant, was placed into a loading buffer consisting of 50 mm Tris-KCl (pH 7.2) with 100 μm of H_2_DCF-DA and fluorescence emission was immediately measured by the LUMIstar microplate luminometer (BMG Labtech, Ortenberg, Germany) at excitation wavelength, 484 nm, and emission wavelength, 525 nm.

### Measurement of ATP concentration via luminescence

ATP concentrations were measured using a luciferin-based ATP assay (Invitrogen, Carlsbad, CA, USA). Similar to H_2_O_2_ detection, citrus leaf discs were placed in the 96 well plate at the bottom of the well except only six leaf discs were used in this assay. The ATP detection buffer was then added into the wells as per the manufacturer’s protocol and the plate was immediately placed in the LUMIstar microplate luminometer for luminescence measurements (BMG Labtech, Ortenberg, Germany).

### Total RNA extraction

Total RNA was extracted from Las-infected and non-infected grapefruit, sweet orange, sour orange and lemon. Sample leaves were quickly frozen in liquid nitrogen and ground to a powder using an autoclaved mortar and pestle. Total RNA was performed as described by Pitino *et al.*^48^

### Real-time quantitative reverse transcription

The SYBR Green FastMix (Quantabio, Beverly, MA, USA) real-time quantitative reverse transcription (RT-qPCR) assay was used to determine the gene expression level of *RBOH*, *APX*, *CAT*, *SOD* for the H_2_O_2_, and both *CATPb* and *MATPb* for ATP (Table 1).

**Table 1:** RT-qPCR primers

*Gene name*	*Primer sequence 5′–3′*	*Reference*
*UPL7*	F: CAAAGAAGTGCAGCGAGAGA	78
	R: TCAGGAACAGCAAAAGCAAG	
*CsRboh*	F: CCCTCGGCTTATAAATGCAA	This study
	R: CAAAAGGCATTGAACCCAGT	
*CsCAT*	F: CCCCCTTCTTTACCACCAAT	This study
	R: TGGGTGACCTCAAAGAAACC	
*CsAPX*	F: GAGGCAAGTCTTTGGTGCTC	This study
	R: GGAAAACAGGGTCATCCAGA	
*CsSOD*	F: GGAATGACATCCCCATCATC	This study
	R: TTGGGTTCGCCTAAATTCAC	
*CsCATPb*	F: TATCCGTATTTGGCGGAGTC	This study
	R: CCATAGTTAGGGCCGTCAAA	
*CsMATPb*	F: CCCTGGCAAGTATGTGGAGT	This study
	R: ATGCAAACCGTGCAACATTA	

SYBR real-time PCR amplifications were performed in a Eppendorf's Mastercycler^®^ ep realplex (Eppendorf, Hauppauge, NY, USA) as previously described.^48^

The UPL7 (ubiquitin protein ligase 7), which was identified as superior reference gene, was selected along with the primers in Table 1 for the RT-qPCR analysis. Expression levels of the target genes were normalized using the following model for the real-time RT-qPCR experiments. The relative expression ratio (*R*) of a target gene is calculated through the *E* and crossing points (CP) deviation of a sample versus a control and expressed in comparison to the reference gene UPL7.^53^

$$Ratio=\frac{{\left(E_{target}\right)}^{\Delta CP_{target}(control-sample)}}{{\left(E_{ref}\right)}^{\Delta CP_{ref}(control-sample)}}.$$

## Results

### Las infection is associated with H_2_O_2_ accumulation in leaf tissue.

In plants, H_2_O_2_ has a dual role as both toxic byproduct of normal cell as well as important signal transduction molecule. When subjected to pathogen attack, H_2_O_2_ production is increased in plants.^54–57^ To investigate the role of H_2_O_2_ in the defense response against Las, we compared Las-symptomatic leaves to non-infected, healthy leaves from four different citrus varieties. The Las-infected leaves were found to produce fluorescent measurements of ~400–500 counts per second (CPS) compared with the healthy controls, which measured ~100 CPS for the same four citrus varieties (Figures 2a–d). Thus, the level of H_2_O_2_ in the Las-infected leaf discs was significantly higher than the non-infected leaf discs (Student’s *t*-test, *P*<0.05), representing an increase of approximately four- to fivefold in infected versus non-infected leaf discs.

### ATP levels increase in Las-infected leaf tissues

Both healthy and infected citrus leaves produce ATP, as it is the main energy source for a majority of cellular functions. We tested both healthy and Las-infected citrus leaves for ATP production using a luciferin-based assay and found a significant increase in ATP accumulation in the four different Las-infected citrus varieties tested compared with their non-infected counterparts (Figure 3). In particular, luminescence measurements were ~15 000 CPS in Las-infected symptomatic leaves. This increased ATP level was relatively consistent and highly significant among the replicates (Student’s *t*-test, *P*<0.05).

### ATP/H_2_O_2_ concentrations directly correlate with HLB symptom severity

To determine whether a relationship existed between ATP, H_2_O_2_ and the degree of symptom severity, we selected Las-infected Duncan leaves and divided them into four different categories based on the severity of their symptoms (Figure 4a). Category 1 encompassed leaves with only very mild HLB symptoms, whereas category 4 contained leaves with severe symptomology. Leaves from each of the four categories were tested for H_2_O_2_ in addition to ATP concentration. These data demonstrated that H_2_O_2_ accumulation increased as the severity of HLB symptoms intensified in the leaves (Figure 4b), indicating a possible role of H_2_O_2_ in causing local tissue damage and thereby the yellowing symptoms. Likewise, ATP levels increased as disease symptoms became more pronounced (Figure 4c). Overall, the ATP and H_2_O_2_ levels correlated with symptom severity, with each of the four categories showing an increase compared with the previously defined category.

### Las infection alters the expression of H_2_O_2_ and ATP-related genes in symptomatic citrus leaves

Exposure of plants to biotic and abiotic stress induces production of ROS, which can lead to oxidative damage to the plant. Therefore, plants with high levels of antioxidants show greater tolerance to this oxidative damage. In this study, we measured the gene expression levels of the key enzymatic antioxidants involved in ROS detoxification: *APX*, *CAT* and *SOD.* In our study, *APX*, *CAT* and *SOD* were downregulated in Las-infected citrus leaves compared with the non-infected leaves (Figure 5). In particular, the transcription level of *APX* was down ~30 fold among the four citrus varieties we tested. These important components involved in H_2_O_2_ detoxification were downregulated in all four Las-infected citrus varieties compared with the non-infected citrus plants. *RBOH*, which led to an early increase in H_2_O_2_ and triggered ROS signaling, was upregulated in all Las-infected citrus plants (Figure 5). Moreover, the gene expression of chloroplast and mitochondria ATP synthase beta-subunits were upregulated in Las-infected citrus leaves (Figure 5 cATP and mATP, respectively).

## Discussion

Citrus greening disease (HLB) is one of the most destructive diseases of citrus worldwide because currently there is no cure, and citrus trees that contract the disease die in as little as five years. Las is associated with the disease in most of the citrus growing regions of the world. Las-associated HLB is not only the most prevalent but has also been associated with increasing economic losses to citrus production worldwide.^1,2^

Identification of the host responses after Las infection is critical for understanding the process involved in the HLB disease development and for the identification of efficient disease management practices. Comparisons of transcription and protein expression studies have demonstrated that various innate immunity components are activated by *Candidatus* *Liberibacter *species.^9,58,59^ In fact, one study demonstrated that 10% of the genes with significantly altered expression patterns after Las infection were related to plant defense and stress mechanisms.^60^ Moreover, Las infection elicits expression of receptor-like kinases (RLKs),^59,61^ even though these proteins are localized to the surface of cells and should not contact the intracellular Las bacterium. This implies that Las PAMPs may possibly be relocated to the cell surface during the course of infection.^62^ Even the long and variable incubation period associated with HLB symptoms suggests that the plants are fighting the disease,^62^ however, an effective immune response has yet to evolve,^2^ as no resistant citrus seedling trees or scion-rootstock combinations have been identified.

In our study, we measured one of the key components of the plant defense response, H_2_O_2_, because it is the most stable ROS molecule and it can pass through membranes. A comparison of four different citrus varieties showed a higher level of H_2_O_2_ in Las-infected versus non-infected plants, indicating an active production of H_2_O_2_ by the plant in response to Las. In our analysis, the increased level of H_2_O_2_ was positively correlated with the overexpression of *RBOH*, which is responsible for mediating ROS production and initiating a long-distance, systemic ROS wave to induce basal resistance, innate plant immunity and systemic acquired resistance.^63–65^ Because an increase in H_2_O_2_ production can result in significant damage to the cells, an antioxidant defense system that detoxifies H_2_O_2_ is regulated by the plant, which functions to catalyze the conversion of H_2_O_2_ into H_2_O.^40^ The H_2_O_2_ detoxification system includes enzymatic antioxidants such as superoxide dismutase (SOD), ascorbate peroxidase (APX) and catalase (CAT).^41,42^ Here, *APX*, *CAT* and *SOD* were all downregulated in Las-infected citrus compared with the non-infected citrus, signifying a decreased ability of the plant to eliminate H_2_O_2_. Several other studies coincide with these results. For example, transgenic tobacco BY-2 cells with lower cAPX activity contained higher intracellular levels of H_2_O_2_.^66^ In another study,^67^ APX1-deficient *Arabidopsis* plants showed a collapse of the entire chloroplastic H_2_O_2_-scavenging system. This caused H_2_O_2_ levels to increase and protein oxidation to occur in leaves subjected to moderate light stress, suggesting that the absence of cytosolic APX1 resulted not only in the accumulation of H_2_O_2_ but also in damage to specific proteins in leaf cells.^40^ The second oxidase found to be down-regulated in Las-infected tissue, CAT, is catalytically involved in the dismutation of two molecules of H_2_O_2_ into water and O_2_.^68^ Catalase-deficient barley displayed a leaf bleaching phenotype,^69^ whereas a twofold increase in extractable H_2_O_2_ was found in *cat2* and *cat2 cat3* knockouts in *Arabidopsis*.^70^ The third component, SOD, constitutes the first line of defense against ROS^71^ and was also found to be downregulated in all four citrus varieties analyzed. SOD double mutants in *Arabidopsis* showed a severe albino phenotype with chloroplast development being arrested in young seedlings.^72^

Taken together, the yellowed shoots, chlorosis and damage distribution of plant tissue typical of HLB may be attributed to the increase in ROS production and plant defense genes such as *Rboh*, which initiates the H_2_O_2_ signal, and simultaneous decrease in the activity of the detoxification systems that corresponds with reduced *APX*, *CAT* and *SOD* expression. Interestingly, genes that could provide an essential defense against ROS generated by the infected plant cells were identified in the Las prophage region. These genes, SC2_gp095 (a ROS-scavenging peroxidase) and SC2_gp100 (a putative glutathione peroxidase), were previously shown to be upregulated in planta relative to the insect host.^38^ In this scenario, the level of H_2_O_2_ would increase during Las infection as a result of the plant's defense response, but the activities of the Las peroxidase would mitigate the direct toxicity of the ROS to the pathogen. Instead, the destructive activity of ROS would damage the plant tissue in the absence of the enzymatic mechanisms aimed at ROS detoxification. This may result in significant damage to cell structures, with symptoms such as blotchy mottles, and chlorosis occurring on the leaf surface as consequence of increased production of ROS.

In this study, we also measured the ATP level using a luciferase leaf discs assay of Las-infected and non-infected citrus leaves from four different varieties. Not only was the ATP level consistently higher among Las-infected leaves of the four different citrus varieties compared with their non-infected counterparts, but the ATP accumulation increased as the disease symptoms became more severe in the infected leaves. The alpha- and beta-subunits of the membrane-bound ATP synthase complex are known to bind ATP and ADP, with the beta-subunits contributing to the catalytic sites, whereas the alpha subunits are involved in the regulation of ATP synthase activity.^73^ In Las-infected leaves, the expression of both chloroplast and mitochondria ATP synthase beta-subunits were upregulated over fivefold, indicating that the plant may produce an increased level of ATP as a result of host–pathogen interactions. The ability of Las to manipulate the plant’s production of ATP to create a nutrient-rich environment, thus fueling the bacteria itself, may have evolved from the close-association that Las has formed with citrus over the past 100 years.^62^ In support of this conclusion is the fact that Las deploys effectors that target the mitochondria^35^ and chloroplast.^36^ Las also possesses a functional ATP translocase that would allow the importation of ATP directly from its eukaryotic host, similar to other obligate intercellular parasites like *Rickettsia prowazeki*.^37,74^ In addition, light-driven ATP synthesis in chloroplasts is very similar to respiration-driven ATP synthesis in mitochondria. ATP is synthesized in both organelles mitochondria and chloroplast by catalyzing the formation of ATP from ADP and inorganic phosphate during oxidative phosphorylation and photosynthesis, respectively.^75^ In conclusion, we have revealed a direct correlation between Las infection in citrus trees and the accumulation of H_2_O_2_ and ATP. We hypothesized that citrus leaves begin to accumulate H_2_O_2_ as the host responds to the Las infection. However, the plant’s detoxification system has not been primed to reduce the increased level of H_2_O_2_ produced, which eventually becomes toxic to the leaf tissue, resulting in the yellowing of shoots or the yellow lesions associated with the blotchy mottling symptoms that appear after Las infection. Conversely, Las survives the toxic conditions generated by the H_2_O_2_ using its encoded peroxidase. Overall, the conclusion drawn from this study that Las infection alters the H_2_O_2_ detoxification pathways of its host concurs with recent findings demonstrating that enzymes involved in radical ion detoxification are upregulated in moderately tolerant citrus compared to highly susceptible varieties and suggests that the upregulation of enzymes involved in radical ion detoxification should be considered a critical mechanism for increased HLB tolerance.^76^ In addition, we show that the level of ATP, one of the most important small molecules in living organisms, was higher in Las-infected plants compared with the non-infected plants. This finding is consistent with a recent study suggesting that in the psyllid Las alters the host environment to enhance nutrient availability and increase ATP levels.^77^ This leads to the intriguing possibility that Las may directly or indirectly increase the ATP level in plant tissues as well for subsequent importation through its translocase directly from its host. Thus, information provided in this study has revealed molecular and physiological mechanisms involved in the host response to HLB and potential new targets for control strategies. Moreover, these molecules, which are induced by Las infection, may be good candidates for biomarkers that can track the progression of HLB in infected plants.

## Disclaimer

Mention of trade names or commercial products in this article is solely for the purpose of providing specific information and does not imply recommendation or endorsement by the U.S. Department of Agriculture.
